# Inexpensive Multiplexed Library Preparation for Megabase-Sized Genomes

**DOI:** 10.1371/journal.pone.0128036

**Published:** 2015-05-22

**Authors:** Michael Baym, Sergey Kryazhimskiy, Tami D. Lieberman, Hattie Chung, Michael M. Desai, Roy Kishony

**Affiliations:** 1 Department of Systems Biology, Harvard Medical School, Boston, Massachusetts, United States of America; 2 Department of Organismic and Evolutionary Biology, Harvard University, Cambridge, Massachusetts, United States of America; 3 FAS Center for Systems Biology, Harvard University, Cambridge, Massachusetts, United States of America; 4 Department of Physics, Harvard University, Cambridge, Massachusetts, United States of America; 5 Faculty of Biology and Department of Computer Science, Technion-Israel Institute of Technology, Haifa, Israel; University of Illinois at Chicago, UNITED STATES

## Abstract

Whole-genome sequencing has become an indispensible tool of modern biology. However, the cost of sample preparation relative to the cost of sequencing remains high, especially for small genomes where the former is dominant. Here we present a protocol for rapid and inexpensive preparation of hundreds of multiplexed genomic libraries for Illumina sequencing. By carrying out the Nextera tagmentation reaction in small volumes, replacing costly reagents with cheaper equivalents, and omitting unnecessary steps, we achieve a cost of library preparation of $8 per sample, approximately 6 times cheaper than the standard Nextera XT protocol. Furthermore, our procedure takes less than 5 hours for 96 samples. Several hundred samples can then be pooled on the same HiSeq lane via custom barcodes. Our method will be useful for re-sequencing of microbial or viral genomes, including those from evolution experiments, genetic screens, and environmental samples, as well as for other sequencing applications including large amplicon, open chromosome, artificial chromosomes, and RNA sequencing.

## Introduction

Sequencing has become an indispensible tool in modern microbiology, dramatically changing the resolution and speed of studies of biodiversity [1], evolution [2–6], and molecular biology [7], and improving pathogen surveillance [8] and clinical diagnostics [9,10]. With current technology, hundreds of full megabase-size genomes can be sequenced in a single Illumina HiSeq lane at over 30x coverage, for a cost of about $15 per sample. Thus, the costs of standard library preparation methods, which typically exceed $50 per sample, substantially limit the amount of microbial genome sequencing. Two studies have recently proposed ways to alleviate this limitation [11,12]. Based on similar principles to those proposed by Lamble et al. [12] and in the Illumina Nextera XT kit [13], we developed a library-preparation protocol that achieves further reductions in costs and increases in efficiency. Specifically, we improve on the cost-limiting steps of these protocols by substantially decreasing tagmentation reaction volume (to 2.5μl), replacing bead-based standardization with inexpensive fluorescent standardization, substituting inexpensive third-party PCR reagents, and replacing the bead cleanup step with functionally equivalent but much cheaper beads. The protocol described here costs approximately $750 per 96 samples including consumables, with under 3 hours hands on time and under 5 hours total time.

## Protocol Overview and Important Considerations

**Fig 1:**
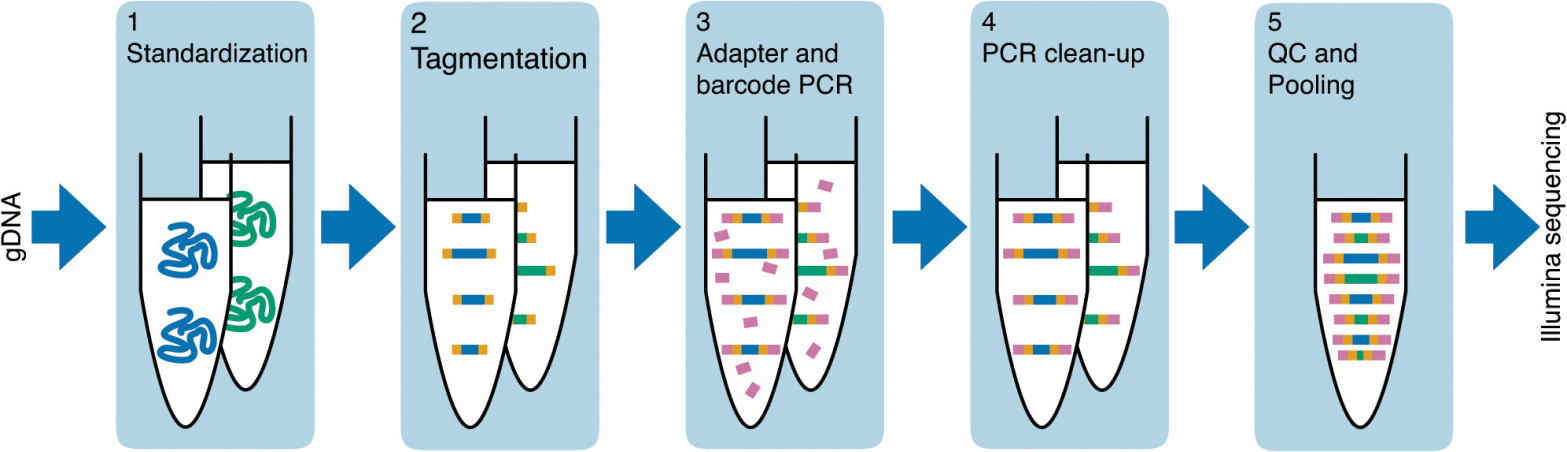
Schematic of library preparation workflow.

Our protocol consists of 5 modules (Fig 1). We assume that the protocol is executed with purified genomic DNA (gDNA) but other types of purified DNA can be used. This protocol is adaptable to any application where template size exceeds read length (e.g. not short amplicon). Since the reliability of the tagmentation reaction (Module 2) is sensitive to the purity of input gDNA [12], we recommend using column-based genomic extraction, such as the Invitrogen PureLink 96-well kit. The cost of consumables per sample is rounded to the nearest $0.25.

### Module 1: Standardization of gDNA concentrations across samples ($0.50/sample, 60 min)

**Fig 2:**
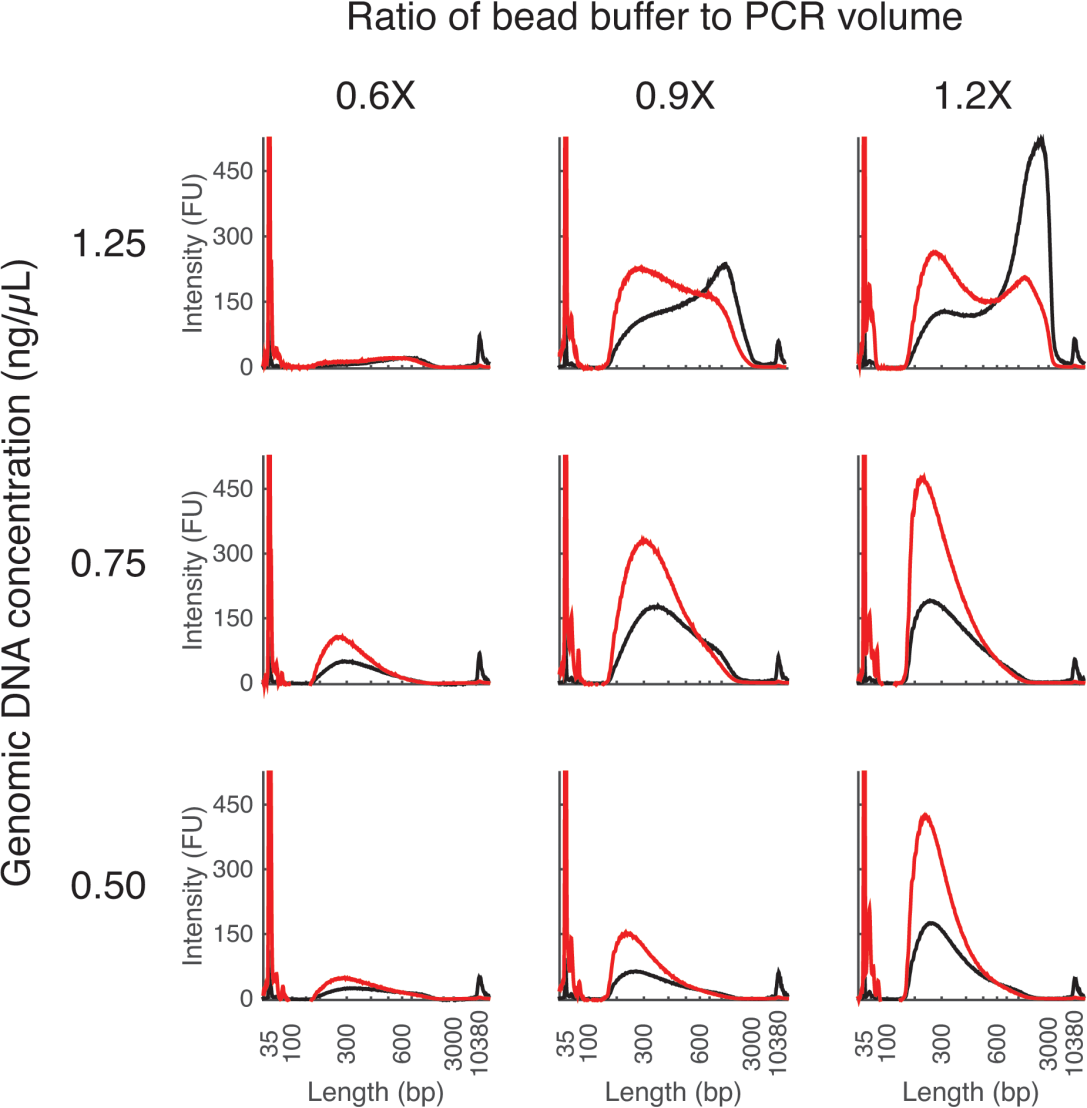
Dependence of fragment size distribution on input gDNA concentration and bead volume. DNA fragment size distribution is affected by starting genomic DNA concentration (rows) as described in Module 1 as well as the relative amount of bead buffer used in PCR clean-up (columns) as described in Module 4. Size distribution is measured by BioAnalyzer and reported in fluorescence units. Data is from *Stenotrophomonas maltophilia* (67% GC, 4.8 Mb genome). At high initial gDNA concentration (1.25 ng/μl) the fragment distribution is right-skewed, though this anomalous peak does not appear to significantly affect sequencing output.

The goal of this module is to standardize the gDNA concentration across samples to achieve uniform reaction efficiency in the tagmentation step (Module 2). Tagmentation is sensitive to the input gDNA concentration and the optimal concentration will vary depending on the organism, DNA type (e.g., genomic versus PCR product), and the DNA extraction method. We found that the optimal initial gDNA concentration may vary depending on the organism and application. In our experience, the optimal concentrations for both Gram-negative (e.g., *Escherichia coli*) and Gram-positive (*Staphylococcus aureus*) bacteria were in the range of 0.5 to 1ng/μl, while for yeast *Saccharomyces cerevisae* it was about 2ng/μl. See “Selecting input gDNA concentration and bead volume for optimal fragment length” and Fig 2 for more information.

We use SYBR Green I to quantify gDNA, which gives sufficiently precise measurements and is markedly cheaper than other dyes. For lower-throughput work, QuBit quantification can also be used. We do not recommend absorbance quantification methods such as NanoDrop because they have lower sensitivity and can be affected by the presence of single-stranded nucleic acids.

### Module 2: Tagmentation ($4.75/sample, 30 min)

**Fig 3:**
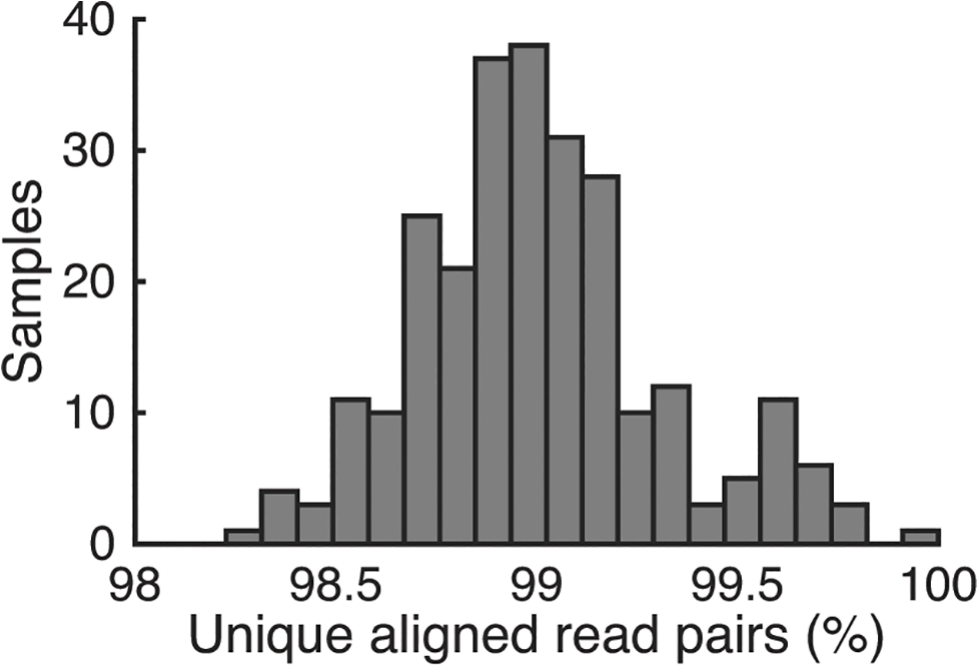
Distribution of the fraction of unique reads over 261 *E*. *coli* samples. Samples had between 0.2 and 2.8 million reads with 90% of samples having over 1 million reads. Raw reads were filtered and then aligned to a reference genome using bowtie2. Unique reads are those that appear only once in the alignment for a particular sample. These are the reads that remain after use of the rmdup tool in samtools. Non-unique reads arise primarily when the same tagmented fragment is amplified during PCR. A low fraction of non-unique reads implies a diversity of fragments after tagmentation, and that errors introduced during PCR will not reach high frequencies.

In this module, the transposase loaded with a part of Illumina adaptors (also referred to as “tagmentation enzyme”) and the tagmentation buffer provided in an Illumina Nextera kit are used to simultaneously fragment gDNA and incorporate sequencing adaptors. We use steps described in the standard Nextera protocol, but with a smaller reaction volume. We have found that tagmentation-reaction volume as small as 2.5μl does not significantly limit the diversity of sequenced DNA for megabase-sized genomes. Specifically, libraries of *E*. *coli* DNA (genome size 4.64Mb) prepared with this protocol typically yield over 98% unique reads (Fig 3). Since each position in the genome is represented on thousands of tagmented DNA fragments, the fraction of false-positive variants created by errors in subsequent PCR amplification (Module 3) are negligible. Larger tagmentation-reaction volumes may be necessary for larger genomes to achieve sufficient library complexity and avoid PCR-induced errors. The final fragment size distribution critically depends on the stoichiometry of gDNA and transposase [14]. Thus, to achieve results consistent across samples, it is essential to accurately standardize input DNA (Module 1) and to thoroughly mix the tagmentation master mix with gDNA. Tagmented DNA, without purification, can be directly used as template for the subsequent PCR step.

### Module 3: PCR-mediated adapter addition and library amplification ($1.50/sample, 75 min)

In this module, PCR is used to incorporate the remaining Illumina adaptor sequences and sample barcodes to tagmented DNA fragments. The adaptors bind fragments to the flow cell [14], and the barcodes allow for multiplexed sequencing. If 96 or fewer samples are pooled on a single lane, we use the Illumina TruSeq primers S501-S508 and N701-N712. For higher multiplexing requirements, we developed custom row and column primers, labeled R09-R36 and C13-C24. These were derived from the TruSeq primers and are compatible with them (S1 Table). Also, Illumina now has additional TruSeq barcodes. When combining the barcodes in this paper with other sets beyond S501-S508 and N701-712, care should be taken to verify that pairs of barcodes remain at sufficiently distant Hamming distances for disambiguation (we recommend at least 3bp).

We substitute the Nextera-provided PCR reagents with KAPA high fidelity library amplification reagents. While we have tested KAPA reagents, in principle any hot start high-fidelity enzyme with low GC-bias amplification should work. Compared to the PCR program recommended in the original Nextera protocol, we recommend a longer initial denaturation to promote inactivation of tagmentation enzyme, shorter extension time to enrich for smaller fragments, and more cycles to increase yield from the smaller tagmentation reaction.

**Fig 4:**
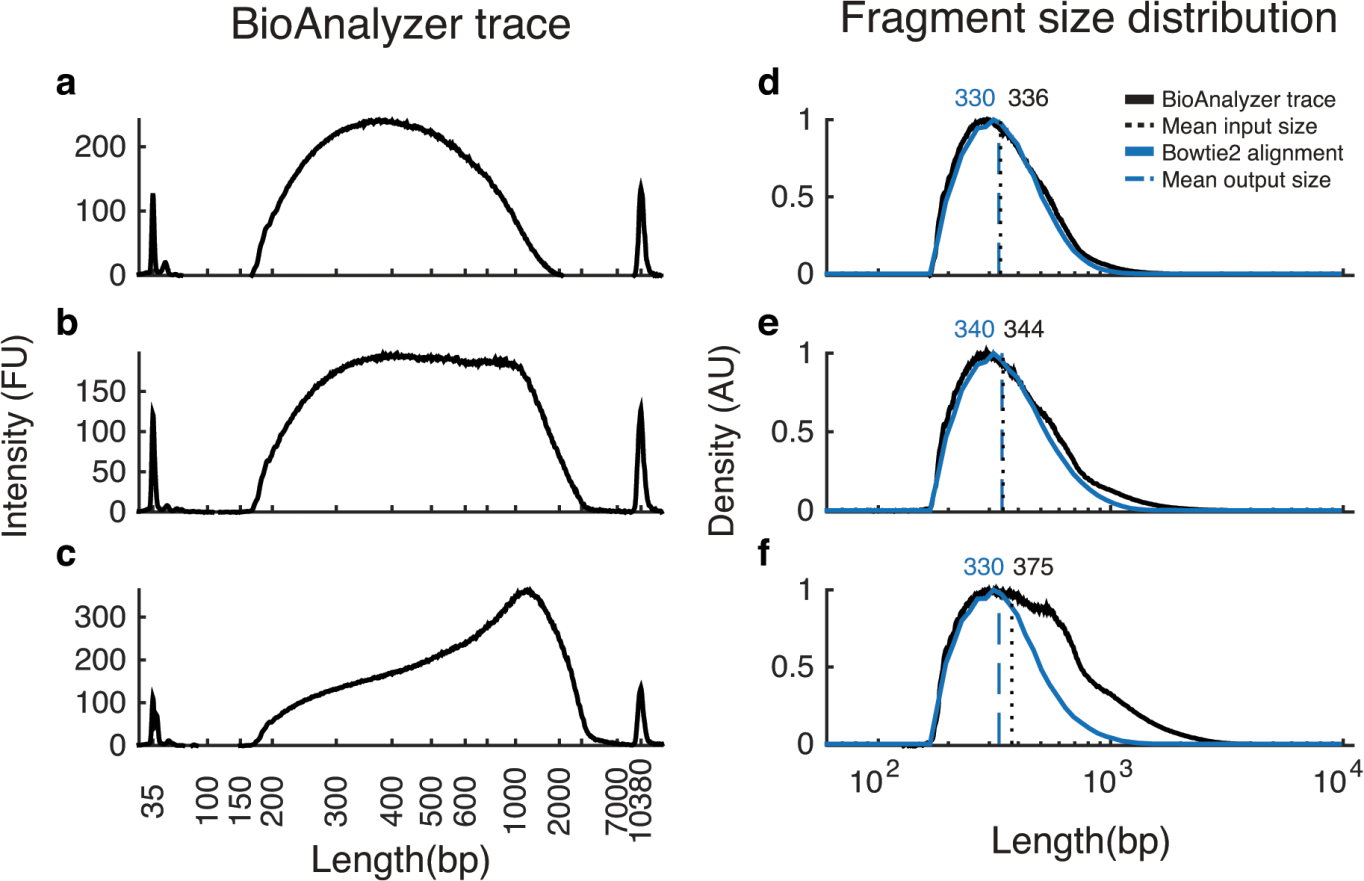
Correspondence between BioAnalyzer traces and the length distribution of aligned reads. Panels A-C show three representative BioAnalyzer traces from three sample preparations of *S*. *maltophilia*. Panels D-F show the corresponding estimated fragment-size distributions (black) and the actual distributions of fragment lengths imputed from alignment to the reference genome (blue). A BioAnalyzer trace *f(x)* shows fluorescence *f* at fragment length *x*. However, we are interested in *n(x)*, the (relative) number of fragments *n* of length *x*. Since fluorescence of a DNA fragment is proportional to its length, *n(x)* ∝ *f(x) / x*. Note that sequencing can be successful despite the presence of apparently very long fragments (which are likely heteroduplexes) in the BioAnalyzer traces (Panels C and F).

An anomalous secondary peak can occur at >1kb in Bioanalyzer traces. While the nature of these apparently long fragments is unclear, they do not appear to substantially affect the Illumina sequencing reaction Nevertheless, they can bias the fragment-size estimation and thereby lead to a decrease in the number and quality of sequenced reads (see Section “Selecting input DNA concentration and bead to sample ratio”). However, in our experience, this never led to a complete failure of the sequencing run (see Fig 4). We also found that various combinations of (a) good mixing prior to tagmentation, (b) higher initial primer concentration, and (c) “reconditioning PCR” (i.e., 3–4 additional cycles with fresh primers and polymerase [15]) can ameliorate this problem.

### Module 4: PCR clean-up and size selection ($0.50/sample, 40 min)

In this module, PCR products are purified with magnetic beads and enriched for fragments of the desired length for sequencing. In lieu of the significantly more expensive Illumina-recommended AmPure XP beads, we use the simple magnetic bead solution with the “MagNA” bead-extraction protocol from [11,16] and Thermo Sera-Mag SpeedBeads. See the “Selecting input DNA concentration and bead to sample ratio” section for more information, Fig 2 for an example. In general, we found that a 1:1 volumetric ratio of sample to beads works well for MagNA as well as AmPure beads. It may also be possible to use a sample purification and standardization kit, such as SequalPrep (Life technologies, ~$0.50/sample).

### Module 5: Library quality control and pooling ($1/sample, 90 min)

**Fig 5:**
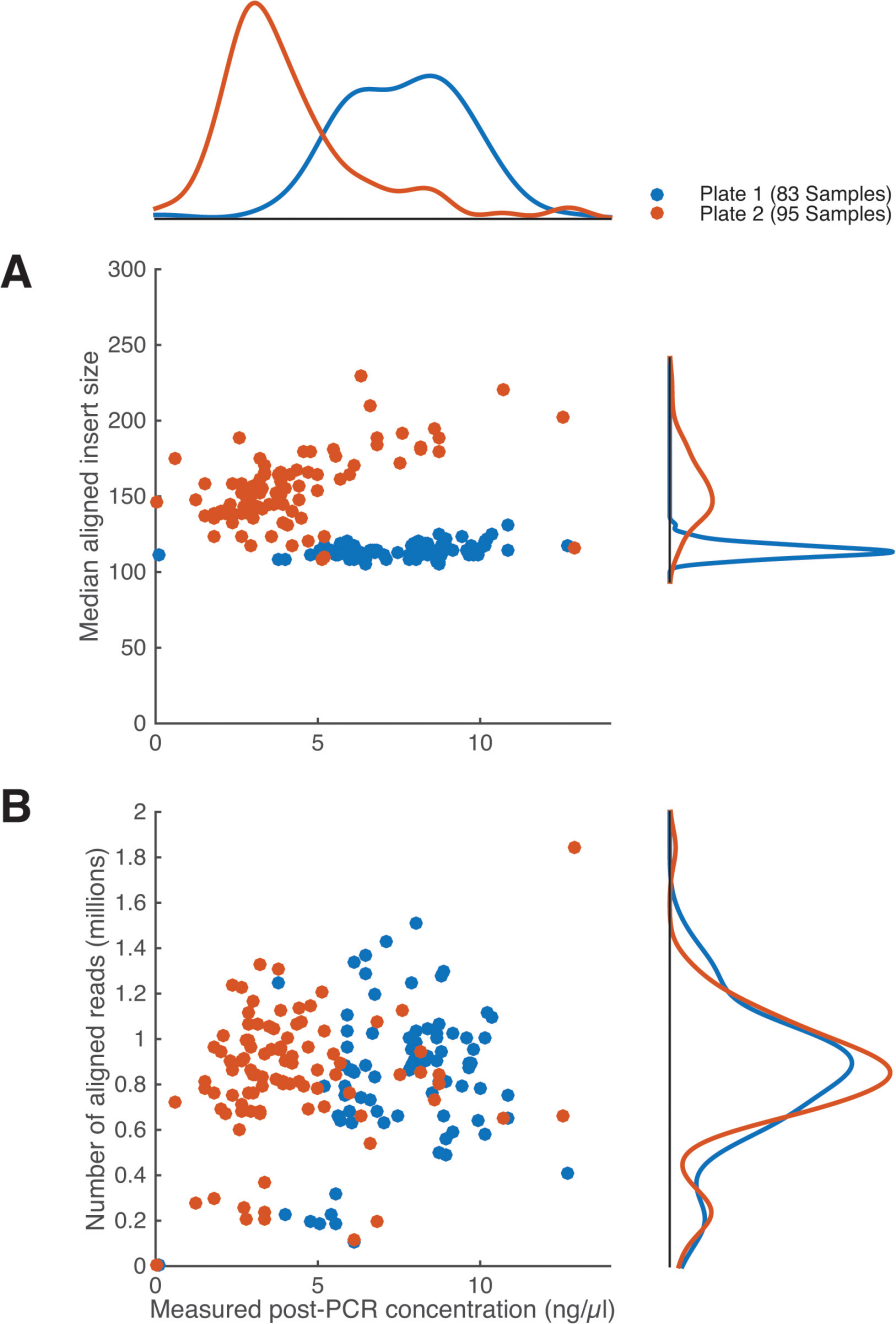
Size and number of aligned fragments as a function of post-PCR DNA concentration. Data is from two plates of *E*. *coli* samples, with 83 and 95 samples per plate (S2 Table). Input gDNA concentrations ranged from 2 to 25ng/μl, and were standardized to 0.5ng/μl. Based on estimated fragment-length distributions, Plates 1 and 2 were pooled in mass ratio 0.8:1. In this preparation, 2 samples (1%) failed to yield libraries, and 17 (10%) produced low, but usable, numbers of reads (between 0.1 and 0.4 million)

In this module, sample concentrations and fragment size distributions are estimated and libraries are pooled. We measure the DNA concentration of each sample fluorescently, as in Module 1; quantification by qPCR is unnecessary at this stage. We discard samples with less than 0.5ng of DNA. Fragment size distribution can be measured with Agilent BioAnalyzer, TapeStation, Bio-Rad Experion, or a number of other devices. While it would be ideal to measure the size distribution of every sample, this is not practical or economically feasible at large scale. Moreover, we found that sample preps from the same 96-well plate typically have similar post-cleanup fragment-size distributions. Thus, we estimate this distribution for a subset of samples (5 to 10). Then, based on individual sample concentrations and the common average fragment length, we calculate the DNA molarity of each sample and pool variable volumes of samples to achieve equimolar concentrations in the pool. Despite the fact that average fragment length can vary across a plate (Fig 5A), Plate 2), calculating molarity based on a few samples results in roughly uniform numbers of reads for ~90% of samples (Fig 5B). For applications that are sensitive to fragment size (e.g. *de novo* assembly), modification of the tagmentation reaction ratios and size-selection cleanup (e.g. via bead-based dual purification, PippinPrep or E-Gel) may be required.

As fragment size distributions often vary more between plates than within plates (Fig 5A), multiple plates should be pooled with equal molarity rather than equal concentration. A final verification of the pooled sample concentration can be useful before sequencing (including qPCR), though most sequencing centers perform this as part of sample quality control.

### Selecting input DNA concentration and bead to sample ratio

For each sample, this protocol produces an adaptor-ligated, barcoded, library of DNA fragments with some distribution of read lengths. The optimal fragment-size distribution depends on the sequencing protocol. To maximize the amount of useful sequenced DNA, most fragments should be longer than the combined length of the sequencing reads and adaptors (e.g. above 338bp for paired-end 100bp sequencing). However, as longer fragments are underrepresented in aligned sequencing reads (Fig 4) and can lower overall cluster density (Illumina Nextera technical notes), fragment length should ideally be kept under 1kb.

We found that the distribution of fragment sizes depends mainly on three parameters: (1) the input concentration of gDNA during tagmentation (output of Module 1); (2) the PCR extension time (Module 3); and (3) the amount of beads during post-PCR clean-up (Module 4). Higher input gDNA concentrations generally result in longer fragments because each tagmentation enzyme makes a single cut [13]. Shorter PCR extension time biases the distribution towards shorter fragments. Finally, higher bead to sample ratios for purification can yield smaller fragments because beads preferentially capture larger fragments [16].

When using a novel DNA source or extraction method, we strongly recommend calibrating the first and the third parameters by dilution series on a representative sample (Fig 2) prior to scaling up the preparation. For example, the initial concentration of DNA might be increased to increase average fragment size.

## Detailed Protocol

This protocol is for the preparation of 96 samples (8 rows x 12 columns) but can be modified for either higher or lower throughput.

### General tips

- We advise cleaning pipettes and your station with DNA-Away (Thermo Scientific 7010) to reduce contamination from the environment and previous samples.
- Many steps call for centrifugation of 96-well plates. These steps are necessary for consistency across wells when to transferring small volumes of liquid and should not be omitted.
- The potential for cross-contamination of samples and primers, in particular, is high. We recommend the use of filter tips, exclusively aspirating samples or primers with fresh tips, and avoiding “blowing out” the pipette.

### Materials and equipment used throughout the protocol

- Sterile DNase-free water
- Filter tips
- Manual multi-channel pipettes. We found less consistent results when mixing with electronic multi-channel pipettes
- DNase-free microfuge tubes, PCR strips, PCR tubes, and PCR 96-well plates
- Centrifuge capable of spinning 96-well plates
- Thermocycler
- (optional) Electronic multi-dispense, multi-channel pipettes
- (optional) 96-well pipette (Liquidator or equivalent)
- (optional) PCR cooler (e.g. Eppendorf 3881)
- (optional) Rubber roller for sealing plates
- (optional) Liquid-handling robot

### Module 1. DNA standardization

#### Goal

Obtain at least 5μl of each sample at concentration 0.5ng/μl (or another chosen concentration)

### Materials and equipment

- gDNA in 96-well plate in range of about 1-10ng/μl
- TE buffer
- 50mL reagent reservoirs
- Seals for 96-well plates (e.g. Microseal B, BioRad MSB-1001)
- 96-well plate with flat transparent bottom for fluorometry (e.g. Corning 3603)
- SYBR Green I (Life technologies S-7563)
- DNA standards in range of 1-10ng/μl (we use those that come with Life Q-33120)
- Plate reader with SYBR-compatible filters

#### Procedure

Note: This procedure assumes gDNA concentration in the range of 1-10ng/μl. If samples cover a different range of concentrations, the procedure should be modified accordingly. We recommend a two step-dilution for samples with a broad range of concentrations.

1. Mix **5μl** of concentrated SYBR Green I with approximately **25mL** of TE in a clean reservoir to make a working dye solution.
  - The same dye solution should be used across all samples and standards.
2. Seal, vortex and centrifuge gDNA (200rcf for 30s).
3. Add **10μl** of gDNA to each well of a fluorometry plate.
  - Recommended: 96-well pipette or multi-channel pipette.
4. In 8 wells of another fluorometry plate, add **10μl** of each DNA standard.
  - We observed little plate-to-plate variability, as long as the same dye solution is used.
5. Dispense **190μl** of dye solution into each well.
6. Incubate the DNA and dye in the dark for 5 minutes, e.g., put in a dark drawer or covered with foil.
7. Read fluorescence on the plate reader using SYBR Green or GFP filters (e.g. Ex:470/40 and Em:520LP).
  - If standard curve is not linear, allow the plate to sit longer and repeat.
  - If the samples are outside the linear range of standards, repeat with a different volume of sample.
8. Based on measured DNA concentration of each sample, calculate the volume of water needed to dilute each sample to 0.5ng/μl.
9. Dispense these volumes of water into a fresh 96-well plate.
  - Recommended: Liquid handling robot
  - If doing manually, a tablet and Pippette-Guide-96 may be helpful (https://github.com/tamilieberman/Pipette-Guide-96).
10. Add **5μl** of gDNA to each well of the new plate.
11. Seal the plate tightly, vortex, and centrifuge (200rcf for 30s).
12. (Optional) Standardized gDNA libraries can be stored overnight at -20°C.

### Module 2. Tagmentation

#### Goal

Mix 1.25μl of TD buffer, 0.25μl TDE1, and 1μl of gDNA in each well. Final total volume per well is 2.5μl. Carry out tagmentation reaction in a thermocycler.

### Materials and equipment

- Standardized gDNA from Module 1
- Nextera TD buffer and TDE1 enzyme (from Illumina kits FC-121-1030 or FC-121-1031)
- 96-well PCR plate (e.g. Bio-Rad MLP-9601)
- Microseal ‘B’ (Bio-Rad MSB-1001)

#### Procedure

Note: All reagents should be kept on ice. The 96-well plate containing samples should also be kept on ice while assembling the mix, and all steps should be done quickly. Small volume reactions can be difficult to work with; do not skip centrifugation.

1. Preheat thermocycler to 55°C. If starting from frozen gDNA, thaw, vortex and spin down gDNA. Thaw TD buffer and TDE1 on ice.
2. Invert TD buffer and TDE1 gently to mix, spin down, and replace on ice.
3. Make the tagmentation master mix (TMM) by mixing **156μl** buffer and **31.2μl** enzyme in a PCR tube. Mix thoroughly by gently pipetting up and down 10 times.
  - Excess volumes enable rapid and even distribution of tagmentation enzyme and buffer. It is essential that all samples receive the same amount of enzyme.
4. Distribute TMM into 8-tube PCR strip with **22.5μl** per tube. Cap and spin down to remove bubbles.
5. Distribute **1.5μl** of TMM per well into all wells of a fresh PCR plate using a multi-channel pipette.
  - Dispense into the bottom of the well and ensure the full amount is dispensed each time.
6. Transfer **1μl** of gDNA into each corresponding well using a manual multi-channel pipette. Mix by gently pipetting up and down 10 times.
7. Cover plate with Microseal ‘B’ and spin down (280rcf for 30s).
8. Incubate in thermocycler for **10min** at **55°C**.
  - Incubation time between 5 and 20 minutes does not affect the results.
9. Place plate on ice. Allow plate to cool before proceeding to Module 3.

### Module 3. PCR-mediated adapter addition and library amplification

#### Goal

Mix 11μl of PCR master mix, 4.4μl of index1, 4.4μl of index2, and 2.5μl of tagmented DNA in each well. Final total volume per well is 22.5μl.

### Materials and equipment

- Tagmented DNA from Module 2
- KAPA HiFi Library Amplification Kit (KAPA KK2611/KK2612)
- Rxxx/Sxxx primers at concentration of 5μM, arrayed in PCR strip (8), S1 Table
- Cxxx/Nxxx primers at concentration of 5μM, arrayed in PCR strips (12), S1 Table
- Microseal ‘A’ (Bio-Rad MSA-5001) or Microseal ‘B’ (Bio-Rad MSB-1001)

#### Procedure

Note: The first 6 steps can be done during the tagmentation reaction (Module 2, step 8). Special care should be taken to avoid primer cross-contamination; only fresh tips should be inserted into the primer stock. It saves significant effort to aliquot the primers into 8- and 12-strip PCR tubes, so that multi-channel pipettes can be used. We recommend strip tubes with individual attached caps or using fresh cap strips each time to minimize potential for cross-contamination. When using Microseal ‘A’, it is important to make sure the plate is fully sealed to prevent evaporation, when using Microseal ‘B’, care must be taken when removing the seal to minimize cross-contamination.

1. Preparation. Thaw indexing primers at room temperature. Invert gently to mix. Spin down. Record which indexing primers you are using. Thaw the KAPA master mix at room temperature. Invert gently to mix.
2. Label one fresh 8-well PCR strip for the row master mix (RMM) and one fresh 12-well PCR strip for the column master mix (CMM).
  - It is easy to accidentally rotate these strips—proper labeling is essential.
  - Place RMM and Rxxx/Sxxx primer strips to the left of the PCR plate.
  - Place CMM and Cxxx/Nxxx primer strips to the top of the PCR plate.
3. Distribute **85.8μl** of 2x KAPA master mix into each empty RMM tube.
4. Distribute **57.2μl** of 2x KAPA master mix into each empty CMM tube.
5. Add **68μl** of each Rxxx/Sxxx primer into the corresponding RMM tube. Mix by pipetting up and down, cap, and spin down.
6. Add **45.6μl** of each Cxxx/Nxxx primer into the corresponding CMM tube. Mix by pipetting up and down, cap, and spin down.
7. Transfer **10μl** of RMMs into each well of the tagmentation plate using a multi-channel pipette, so that each row receives the same Rxxx index.
  - Change tips after every transfer.
  - Make sure that the row number corresponds to the Rxxx.
8. Transfer **10μl** of CMMs into each well of the plate using a multi-channel pipette, so that each column receives the same Cxxx/Nxxx index. Mix by gently pipetting up and down 10 times.
  - Change tips after every transfer.
  - Make sure that the column number corresponds to the Cxxx.
9. Cover plate with Microseal ‘A’ or ‘B’. Spin down (200rcf for 30s).
  - Gently press the seal on each well, especially edge wells before spinning down; this seal is non-adhesive until heat is added.
10. Place plate in the thermocycler.
  - Ensure that the lid is tight and is heated during thermocycling.
11. Run the following program:
  1. 72°C for 3 min
  2. 98°C for 5 min
  3. 98°C for 10 sec
  4. 63°C for 30 sec
  5. 72°C for 30 sec
  6. Repeat steps (3)-(5) 13 times for total of 13 cycles
  7. 72°C for 5 min
  8. Hold at 4°C
12. (Optional) If using Microseal ‘B’, the plate can be left at 4°C overnight.

### Module 4. PCR clean-up and size selection

#### Materials and equipment

- Tagmented and indexed DNA
- 100% ethanol
- Resuspension buffer (10mM Tris-Cl [pH 8.0] + 1mM EDTA [pH 8.0] + 0.05% Tween-20)
- Deep-well 96-well plate for bead purification
- Magnetic beads for DNA purification (2% v./v. Sera-Mag SpeedBeads, 18% w./v. PEG-8000, 1M NaCl, 10mM Tris HCl, 1mM EDTA, 0.05% Tween 20; e.g. according to http://ethanomics.files.wordpress.com/2012/08/serapure_v2-2.pdf)
- 96-well plate magnetic stand (e.g., Life Technologies, Cat. #123-31D).

#### Procedure

Note: It is best to thaw beads at the beginning of Module 1, as it takes time for them to reach room temperature. While at this stage cross-contamination is a much smaller issue, we still recommend fresh tips for each well.

1. Centrifuge the PCR plate at 200rcf for 30 seconds.
2. To resuspend beads, alternate between vortexing and inverting beads for a total of at least 60 sec.
3. Transfer at least **2.5ml** of beads into a reagent reservoir.
4. Using a multi-channel pipette, transfer **22.5μl** of beads into each well of the PCR plate and pipette up and down several times to mix.
  - Pipette into the bottom of wells and ensure that the beads are completely dispensed.
  - Some prefer to purify in a separate deep-well 96-well plate, for easier aspiration. In this case, we recommend first adding 15μl of beads to each well of the deep-well plate, then transferring 15μl of sample into each well and mixing.
5. Incubate at room temperature for **5 min**. DNA is now on the beads.
6. Prepare a fresh batch of 80% ethanol by mixing **10mL** of sterile water and **40mL** of 100% ethanol in a sterile reservoir.
7. Place the plate on the magnetic stand and incubate for **1 min** to separate beads from solution. The solution should become clear.
8. While the plate is on the magnetic stand, aspirate clear solution from the plate and discard. Do not disturb the beads. If beads are accidentally aspirated, resuspend them, wait 1 min, and aspirate again.
9. While the plate is on the magnetic stand, dispense **200μl** of 80% ethanol into each well. Incubate for **1 min** at room temperature.
10. Aspirate ethanol and discard. Do not disturb the beads. If beads are accidentally aspirated, resuspend them, wait 1 min, and aspirate again.
11. Repeat steps 9–10 for a total of 2 washes.
12. Remove any visibly remaining ethanol droplets with smaller pipette tips.
13. Let the plate air dry for **20 min** for residual ethanol to evaporate.
  - This is a good time to take out the gel-dye matrix for the BioAnalyzer in Module 5, to allow it to come to room temperature.
14. Transfer at least **3.5ml** of resuspension buffer to a new reservoir.
15. Take the plate off the magnetic stand. Add **30μl** of resuspension buffer to each well of the plate using a multichannel pipette. Resuspend the beads by mixing 10–15 times.
16. Incubate for **5 min** at room temperature. DNA is now in the solution.
17. Place the plate back onto the magnetic stand and incubate for about **1 min** to separate beads from solution. The solution should become clear.
18. While the plate is on the magnetic stand, aspirate clear solution from the plate and transfer to a fresh 96-well plate. Do not disturb the beads. If beads are accidentally pipetted, resuspend them, wait for the solution to become clear, and repeat.
19. Seal plate and spin down (200rcf for 30s).
20. (Optional) DNA libraries can be stored at -20°C before proceeding to Module 5.

### Module 5. Library QC and Pooling

#### Materials and equipment

- Purified Nextera libraries from Module 4
- High Sensitivity DNA kit for BioAnalyzer (Agilent 5067–4626)
- TE buffer
- 50mL reagent reservoirs
- 96-well plate with flat transparent bottom for fluorometry (e.g. Corning 3603)
- SYBR Green I (Life technologies S-7563)
- DNA standards in range of 1-10ng/μl (we use those that come with Life Q-33120)
- Plate reader with SYBR-compatible filters
- BioAnalyzer (Agilent 2100) or similar DNA fragment-size assay system.

#### Procedure

1. Perform steps 1–7 of Module 1 to quantify DNA concentration across all samples.
  - You may use less DNA and ladder (5 or 8 μl) to conserve sample.
2. Calculate the concentration of each sample. We typically discard samples with low concentrations (< 0.5ng/μl), which would not have enough coverage and would dilute the final concentration of pooled samples.
3. Select a number of samples per batch (96-well plate) for fragment quantification.
4. Prepare High Sensitivity DNA Analyzer chip per manufacturers instructions, load samples onto chip, and perform analysis.
5. Determine if fragment-size distributions in each batch are acceptable. See Figs 2 and 4 for reference and main text for discussion. Calculate sample molarity based on batch-specific average fragment length.
6. Pool acceptable samples in equimolar concentrations.
7. If sequencing in-house, accurate quantification is crucial to achieve optimal cluster density. We recommend using qPCR (KAPA KK4824) and running a BioAnalyzer on the final pooled library.
8. The pooled library should be stored at -20°C.

## Supporting Information

S1 Table R5xx/C7xx primer syntax and primer sequences for R501-R536 and C701-C724. (CSV)

S2 Table Data from two plates of E. coli samples. Plate number; measured post-PCR concentrations of sample; and number, median, standard distribution, and histogram of aligned fragment sizes are provided.
(TXT)
