# Correction: Inexpensive Multiplexed Library Preparation for Megabase-Sized Genomes

**DOI:** 10.1371/journal.pone.0131262

**Published:** 2015-06-18

**Authors:** The PLOS ONE Staff

The abbreviation of the corresponding author's name in the author contributions is incorrect. The correct abbreviation is MMD.

S1 Table and S2 Table files are switched. Please view the correct S1 Table ad S2 Table below. The publisher apologizes for these errors.

## Supporting Information

S1 Table R5xx/C7xx primer syntax and primer sequences for R501-R536 and C701-C724. (TXT)

S2 Table Data from two plates of E. coli samples. Plate number; measured post-PCR concentrations of sample; and number, median, standard distribution, and histogram of aligned fragment sizes are provided.
(CSV)
